# The Activation of Effect Codes in Response Preparation: New Evidence from an Indirect Priming Paradigm

**DOI:** 10.3389/fpsyg.2012.00585

**Published:** 2012-12-31

**Authors:** Michael Ziessler, Dieter Nattkemper, Stefan Vogt

**Affiliations:** ^1^Department of Psychology, Liverpool Hope UniversityLiverpool, UK; ^2^Department of Psychology, Humboldt University of BerlinBerlin, Germany; ^3^Department of Psychology, Centre for Research in Human Development and Learning, Lancaster UniversityLancaster, UK

**Keywords:** action planning, effect anticipation, ideomotor theory, motor control

## Abstract

Evidence for the anticipation of environmental effects as an integral part of response planning comes mainly from experiments in which the effects were physically presented. Thus, in these studies it cannot be excluded that effect codes were activated during response preparation only because the effects were displayed as external stimuli before response execution. In order to provide more clear-cut evidence for the anticipation of response effects in action planning, we performed a series of three experiments using a new paradigm, where displaying effect codes before the response was avoided. Participants first learned arbitrary effects of key-pressing responses. In the following test phase they were instructed to execute a response only if a Go stimulus was presented after a variable stimulus onset asynchrony (SOA). The Go stimulus was either compatible or incompatible with the effect, but independent of the response. In Experiment 1 we tested the paradigm with two responses and two effects. We found a significant compatibility effect: If the Go stimulus was compatible with the response effect, responses were initiated faster than in incompatible trials. In Experiment 2 response effects were only present in the acquisition phase, but not in the test phase. The compatibility effect disappeared, indicating that the results of Experiment 1 were indeed related to the anticipation of the forthcoming response effects. In Experiment 3 we extended this paradigm by using a larger number of stimuli and response alternatives. Again we found a robust compatibility effect, which can only be explained if the effect representations are active before response execution. The compatibility effects in Experiments 1 and 3 did not depend on the SOA. The fact that the Go stimulus affected response preparation at any time indicates that the role of effect anticipation is not limited to response selection.

## Introduction

Human motor behavior, when it is not reflexive, is typically carried out to achieve goals. When we plan a movement we normally have an environmental or sensory effect in our mind that we want this movement to produce. This can either be in form of the physical movement itself, like in floor exercises or in dancing, or a more distal effect like the creation of a new environmental state, or the avoidance of an unpleasant situation. This leads to the question how the environmental effects, or their representations, are involved in the planning and control of movements.

Early theories of movement control indeed considered the anticipation of the sensory effects of a movement as a prerequisite to perform the movement. By randomly executing a movement, the performer learns which sensations are connected with this movement. The re-activation of the sensations will then produce the same movement, or at least a corresponding movement tendency (Herbart, 1824; Lotze, 1852; Harleß, 1861; James, 1890; Münsterberg, 1888). James (1890) called this the ideomotor principle. More modern versions of the ideomotor principle follow this suggestion by assuming that voluntary responses or actions are centrally represented by the sensory feedback that they produce, i.e., by their effects (Greenwald, 1970; Prinz, 1983, 1997; Hommel et al., 2001; Hommel, 2009; see also Stock and Stock, 2004; Pfister and Janczyk, 2012 for an overview).

Also other theories of motor control consider the anticipation of action effects as an important component of the control process, e.g., Schmidt’s (1975, 1988) Schema Theory and the more recent concept of forward models in motor control (Davidson and Wolpert, 2005; Wolpert and Flanagan, 2009). These theories basically assume that that the sensory effects of a selected motor response are anticipated in order to allow for the internal testing of the programmed response, the monitoring of execution, and the related error detection and correction.

Thus, theoretically it has been well established that action effects play a crucial role in the selection, preparation, and execution of motor actions. This theoretical emphasis has led to numerous experiments trying to find evidence for the anticipation of effects for the selection, internal test, and monitoring of motor responses (see Nattkemper et al., 2010; Shin et al., 2010 for reviews). However, it should be noted that a considerable part of this evidence comes from experiments in which the effects were presented prior to response execution. Obviously, an experimental setting in which response effects are physically present in the environment does not have high ecological validity. Under normal conditions the appearance of the effect in the environment would indicate the successful completion of the response but not trigger its execution. The fact that advance presentation of an effect facilitates the response could therefore be an artifact of the experimental situation. It could be that effect representations are only activated because of the external stimulation and that participants only use the effect information to select and prepare the required response because it is already available.

In particular, this criticism applies to all paradigms in which the response effects were used as imperative stimuli, or where the response effects were presented together with the imperative stimuli. A first paradigm of this kind is based on the theoretical assumption that the acquisition of goal-directed actions follows a two-stage process (Lotze, 1852; Hommel, 1996; Elsner and Hommel, 2001). In the first stage, randomly executed movements are associated with their effects. The association is assumed to be bi-directional, so that in a second stage the activation of effect representations automatically leads to the activation of the corresponding movement that is needed to produce the effect. In their experiments, Elsner and Hommel (2001) first let their participants execute two key-presses that were followed by a low pitched or a high pitched tone. In the second phase of the experiment the tones were used as imperative stimuli in a forced-choice task. Responses to the tone stimuli were faster if the tone-response assignment corresponded to the response effect relation from the first part of the experiment. Similarly, if the second phase of the experiment was a free-choice task, participants responded to a given tone more often with the response that produced this tone beforehand, rather than with the alternative response (see also, Pfister et al., 2011).

In a second paradigm to which our criticism also applies the effects were presented simultaneously with the imperative stimulus, and participants were instructed to ignore them. For example, in some of our own experiments (Ziessler and Nattkemper, 2002, 2011; Ziessler et al., 2004) we adapted the flanker task (Eriksen and Eriksen, 1974) to investigate the integration of effect anticipation in action planning. In an initial acquisition phase participants performed key-pressing responses to letters that were followed by other letters as effects. In the test phase participants performed the same forced-choice reaction task, but now the effect letters were presented as flanking stimuli on both sides of the imperative stimulus. Responses were facilitated if the flanking letters were the effects of the correct response to the imperative stimulus.

A third paradigm, which is less affected by our criticism, provides evidence that responses are facilitated if there is an overlap between features of the response and features of the effect (Greenwald, 1970; Kunde, 2001, 2003; Kunde et al., 2004). The experiments by Kunde and collaborators show, for example, that spatial compatibility between the location of the response and the location of the effect in a forced-choice reaction task facilitates the response. Also, if the intensity of the response (e.g., soft or forceful key-presses) was compatible with the intensity of the effect (e.g., loudness), or the durations of responses and effects corresponded to each other, responses were facilitated. Kunde and collaborators interpreted their findings of response effect compatibility, in analogy to activation models of stimulus-response compatibility (Kornblum et al., 1990), via the assumption that the feature overlap between responses and their effects leads to a facilitation of the responses. In line with the ideomotor principle, if there is dimensional overlap between the response and the effect, features of the effect can directly activate features of the response. The advantage of this paradigm is that the effects are not physically presented before response execution. However, the evidence is limited to situations in which responses and their effects show a dimensional overlap. There are indeed instances outside of the laboratory setting for such dimensional overlaps, for example, the correspondence between the duration of a key-press and the duration of a tone, or between the force of a response and the force of the effect. But in many other instances our movements do not exhibit any feature overlap with the distal effects that they produce in the environment. For example, the movements that we execute to bring up the letters on the computer screen have actually nothing in common with the letters; the movement to turn a light switch has no features in common with the light that is switched on.

The most convincing evidence for effect anticipation so far comes from experiments that adopted a fourth paradigm. Kunde et al. (2002) instructed their participants to prepare a response, but in a number of critical trials they had then to switch to another response. This switch could be performed faster when both the originally cued response and the switched response produced the same effect. Kunde et al. (2002) argued that their results show that effect codes become endogenously activated in response preparation and that the activated effect representations are capable of also activating other responses that produce these effects. To our knowledge, however, the advantage of switching between responses that produce the same effect, as compared to responses producing different effects, was only shown where tones were used as effects. The production of tones might be a special case, and so it is still unclear if those findings could be generalized to other response effects.

In summary then, in a considerable number of the available experiments on effect anticipation, the response effects themselves were used as imperative or as flanker stimuli, which compromises the interpretation of the results (see above). The two other experimental paradigms used to date are less affected by our circularity argument, but generalization of those findings is limited to situations where responses and effects show dimensional overlap (third paradigm), or, at least to date, to tones as environmental effects (fourth paradigm).

With the present series of experiments we devised a new paradigm, which neither includes the physical presentation of response effects nor relies on a feature overlap between responses and their effects. Basically, we used an indirect priming paradigm in which a stimulus presented during response preparation has the potential to interact with the effect of the response, provided the response effect is indeed activated at this point in time. The experiments started with an acquisition phase, where participants learned that their responses produced particular, arbitrarily assigned effects. The following test phase was designed as a Go/No-Go task. Participants were instructed to prepare a response, but they should only perform the response after a Go stimulus was presented. The identity of the Go stimuli had no relationship to the responses, but they were either compatible or incompatible with the response effect. Because the Go stimuli do not provide any information regarding the required response, they can only affect the response via their relationship to the response effects. Therefore, if the effect compatibility of the Go stimuli would affect the reaction times (RTs), this would convincingly demonstrate that effect codes have functional relevance for the preparation of the responses.

The aim of the first experiment was to test this paradigm using just two responses, two effects, and two Go stimuli. We predicted that effect-compatible Go stimuli would support the activation of the response-related effect code and that this would, in turn, facilitate the response, in comparison to effect-incompatible Go stimuli. There are at least two different hypotheses how a facilitation of the effect anticipation could result in faster responses. If effect codes were used for response selection (Elsner and Hommel, 2001; Hommel et al., 2001; Kunde et al., 2002) an early activation of the effect representation should lead to a faster response. If the activation of effect representations is part of the preparation of an already selected response (Schmidt, 1975, 1988; Ziessler and Nattkemper, 2011), then the additional activation of the effect by the compatible Go stimulus would shorten the process of response preparation, and consequently reduce the RTs. In an attempt to distinguish between these two hypotheses, we varied the stimulus onset asynchrony (SOA) between the imperative stimulus and the Go stimulus in the test phase. The first hypothesis would predict stronger compatibility effects for shorter SOAs, whereas the second hypothesis would either predict the opposite effect, indicating an increasing role of effect anticipation in later stages of response preparation, or no interaction between compatibility and SOA.

With the second experiment we wanted to assess whether the presence of the response effects in the test phase was crucial for the observed differences between effect-compatible and effect-incompatible trials, or whether the association between responses and effects, which was established during the practice phase, would be sufficient for compatibility effects to emerge in the test phase, even if the effects were no longer present. Disappearance of the compatibility effect would indicate that, in our paradigm, effects are only anticipated if they actually continue to appear after the response. Such a result would further validate Experiment 1 by showing that the observed effect was indeed due to the processing of the effect information during response preparation, and not due to the imperative stimuli or Go stimuli and their assignment to the responses.

In Experiment 3 we explored a wider range of SOAs than in Experiments 1 and 2. In addition, we used a larger number of response alternatives in order to extend the duration of response preparation, and introduced a second type of effect-incompatible Go stimuli, which were not related to any of the response effects. The latter variation was intended to provide more information about facilitating and inhibiting mechanisms contributing to the effects of compatible and incompatible Go stimuli on response preparation.

## Experiment 1

**Figure 1:**
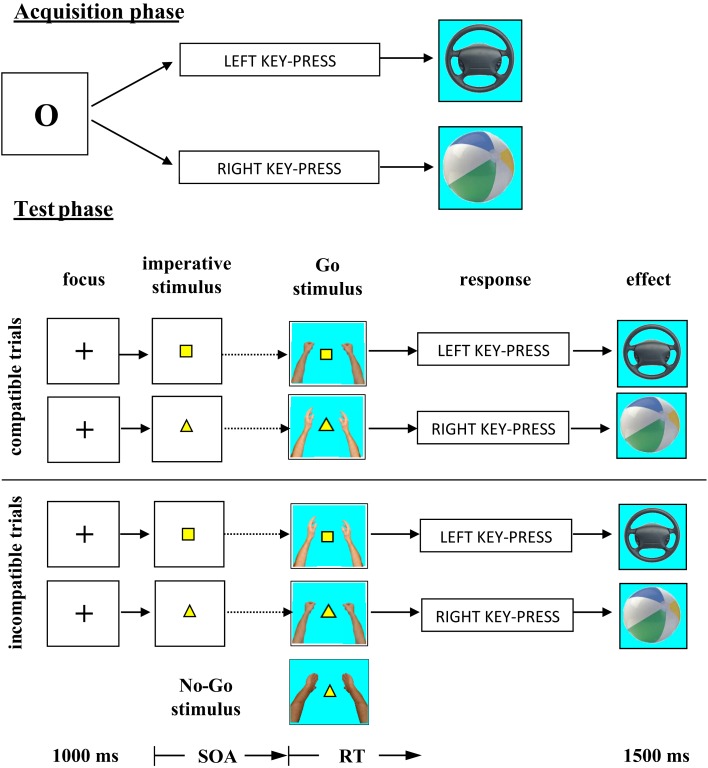
**Material and design used in Experiment 1**. The SOA was 0, 100, or 200 ms.

The acquisition phase consisted in a free-choice task. According to previous experiments, the free-choice task provides optimal conditions for response effect learning (e.g., Elsner and Hommel, 2001; Herwig et al., 2007; Pfister et al., 2010). Participants learned that the execution of each of two key-presses was followed by a particular effect; one of the responses produced a picture of a car steering wheel on the computer screen, the other response a picture of a beach ball. In the test phase, an imperative stimulus defined which of the responses participants should execute. Response execution was only to be carried out after presentation of one of the two Go stimuli. The Go stimuli consisted either of a picture of hands in the posture of catching a beach ball or hands in the posture of holding a steering wheel (Figure 1). Obviously, the hands in the posture of holding a steering wheel were compatible with the steering wheel and incompatible with the beach ball and vice versa. The question was: would the effect compatibility of the Go stimuli affect the RTs? Effect-compatible Go stimuli should support the activation of the effect codes, effect-incompatible Go stimuli should interfere with the activation of the effect codes.

### Method

#### Participants

Forty-two undergraduate students from the Department of Psychology at the University of Sunderland served as participants. All students were first-year students and received course credit for their participation. Their mean age was 21.3 years (SD = 4.9). Thirty-five of the participants were female, nine male. Participants had either normal or corrected to normal vision. Ethical approval was obtained from the departmental ethics committee.

#### Material and apparatus

Stimulus presentation and response recording were controlled by a standard PC. The computer was situated in a sound-proof booth. In the acquisition phase of the experiment the letter O was presented in the middle of the computer screen. Responses consisted of a key-press with either the left or right index finger. Participants were instructed to use the X-key of a standard QWERTY keyboard with their left index finger and the M-key with their right index finger. Both fingers were located on the corresponding keys throughout the experiment. After a left key-press a picture of a steering wheel appeared in the middle of the computer screen. After a right key-press a picture of a beach ball was presented as response effect (see Figure 1).

In the test phase, a yellow square required a response with the left index finger and a yellow triangle a response with the right index finger. Two hands in the posture of holding a steering wheel and two hands in the posture of catching a beach ball were used as Go stimuli. A No-Go trial was indicated by two hands with the palms turned outwards (see Figure 1 for illustration of the Go and No-Go stimuli). The high similarity between Go and No-Go stimuli forced the participants to identify these stimuli if they did not want to produce a high number of false alarms in the No-Go trials.

#### Design and procedure

The experiment was divided into two phases: an acquisition phase and a test phase. In the acquisition phase the participants performed a free-choice reaction task. Upon presentation of an “O” in the middle of the screen participants were asked to perform either a left or a right key-press depending on their choice. They were instructed to use both key-presses with about the same frequency. The key-press deleted the “O” from the screen and triggered the presentation of the response effect. After a left key-press the steering wheel appeared on the screen whereas the right key-press was always followed by the beach ball. The effect stimuli remained on the screen for 1500 ms. After an inter trial interval (ITI) of 500 ms the next trial began with the presentation of the “O.” The acquisition phase consisted of 100 trials. After 50 trials and at the end of the acquisition phase participants were informed about the frequency of their use of each key.

The subsequent test phase was designed as a forced-choice reaction task. Each trial started with the presentation of a “+” sign for 1000 ms followed by the imperative stimulus, either a square or a triangle, which determined the response. Participants were instructed to withhold the response until the presentation of a Go stimulus, after which they should execute the correct response as quickly as possible. In case of the No-Go stimulus participants should not respond. The pictures of the two hands constituting the Go or No-Go stimulus appeared on the left and right sight of the imperative stimulus. The SOA between the onset of the imperative stimulus and the onset of the Go stimulus was either 0, 100, or 200 ms. Immediately after a correct response, the effect stimulus assigned to that response replaced the imperative stimulus and the Go signal. As in the acquisition phase, the effect stimulus remained on the screen for 1500 ms and, after an ITI of 500 ms, the next trial started with the “+” sign. In the case of an incorrect response, instead of the effect stimulus appearing, the word “incorrect” was presented for 1500 ms. No-Go trails were terminated after 2000 ms by the presentation of a blank screen for 500 ms. If participants performed a response in No-Go trials the response was followed by a written reminder that they should not respond in such trials.

The most important independent variable of the experiment consisted of the construction of the Go trials. Both imperative stimuli could be followed by both Go stimuli. This meant there was no fixed relationship between the two Go stimuli and the two responses. However, because there was a fixed relationship between responses and effect stimuli, in half of the Go trials the Go stimuli were compatible with the response effect and in the other half incompatible (cf. Figure 1).

Altogether the test phase consisted of 360 trials, divided into six blocks of 60 trials with short rest periods between blocks. Of all trials, 80% were Go trials and 20%were No-Go trials. The three SOAs were applied with equal frequencies to Go and No-Go trials. All experimental conditions were randomly mixed across the test phase. Responses and RTs were measured from the onset of the Go stimulus. Figure 1 summarises the experimental procedure. Including the acquisition phase the complete experiment lasted about 50 min.

### Results

The aim of the acquisition phase was plainly to familiarize the participants with the response effects. There was no further data analysis. The data of interest regarding our research question were collected in the Go trials of the test phase. Only RTs from correct responses were taken into account. RTs longer than 2000 ms were considered as outliers. Overall, only 3.6% of all responses in Go trials were erroneous responses or outliers.

Individual mean RTs for each participant were calculated depending on the SOAs and the effect compatibility of the Go stimuli. The individual means were subjected to a 3 (SOA) × 2 (compatibility) repeated-measures Analysis of Variance (ANOVA). In this and all following analyses, sphericity was tested for all repeated-measures factors with more than two levels. If sphericity could not be assumed, the degrees of freedom and subsequently the *p*-values were corrected using the Greenhouse–Geisser correction.

**Figure 2:**
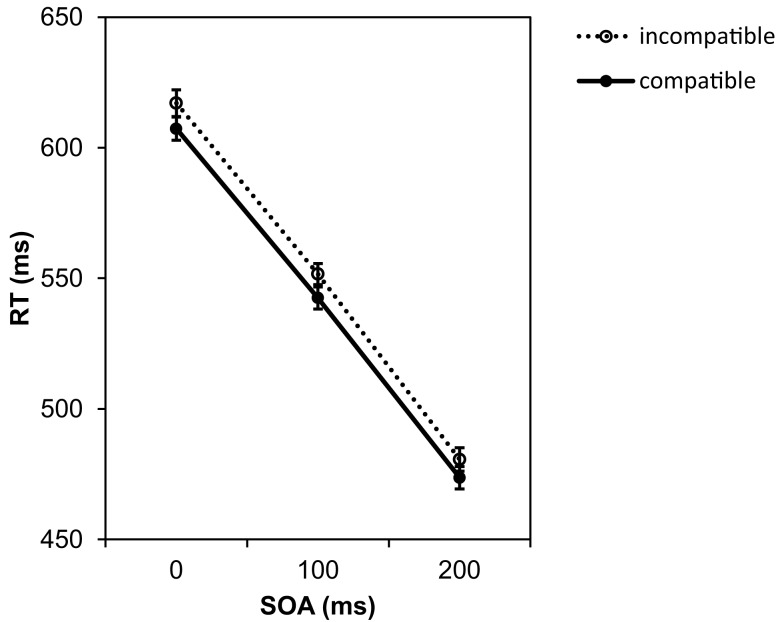
**Mean reaction times depending on SOA and compatibility conditions in Experiment 1**. Error bars in this and all following graphs represent the standard error of the means calculated for the within-participant design following the procedure suggested by Cousineau (2005).

Figure 2 presents the mean RTs for all experimental conditions. The ANOVA yielded a significant main effect of the SOA, *F*(2, 82) = 308.71, *p* < 0.001, $\eta_{p}^{2}=0.88.$ Whereas at the 0 ms SOA the mean RT was 612 ms, at the longest SOA of 200 ms the RTs were about 135 ms shorter. This indicates that participants used the SOA interval to prepare the response. More importantly, there was also a significant compatibility effect, *F*(1, 41) = 5.14, *p* = 0.029, $\eta_{p}^{2}=0.11.$ If the Go stimulus was compatible with the effect of the to-be-prepared response, RTs were on average 9 ms faster than in the incompatible condition. According to Cohen (1988), $\eta_{p}^{2}=0.11$ indicates a medium effect size. This is first evidence for an activation of effect codes during preparation of the motor response. The interaction between both variables was not significant, *F*(2, 82) = 0.06, *p* = 0.946, $\eta_{p}^{\text{2}}=0.001.$

Given numerically small compatibility effect we checked carefully if this difference could be explained by a speed-accuracy trade-off. However, this was not the case. An ANOVA conducted on the number of correct responses only found a significant effect of SOA, *F*(2, 82) = 11.77, *p* < 0.001, $\eta_{p}^{2}=0.22.$ The number of correct responses increased with the prolonged SOA from 94.5 to 97.2%. However, there was no effect of compatibility, *F*(1, 41) = 0.56, *p* = 0.460, $\eta_{p}^{2}=0.01.$ On average, 95.9% of the responses in compatible trials were correct. For incompatible trials the figure increased to 96.3%. There was also no interaction between SOA and compatibility, *F*(2, 82) = 1.34, *p* = 0.268, $\eta_{p}^{2}=0.03.$ Therefore, we can exclude a speed-accuracy trade-off as a cause for the compatibility effect observed in the RT data.

**Figure 3:**
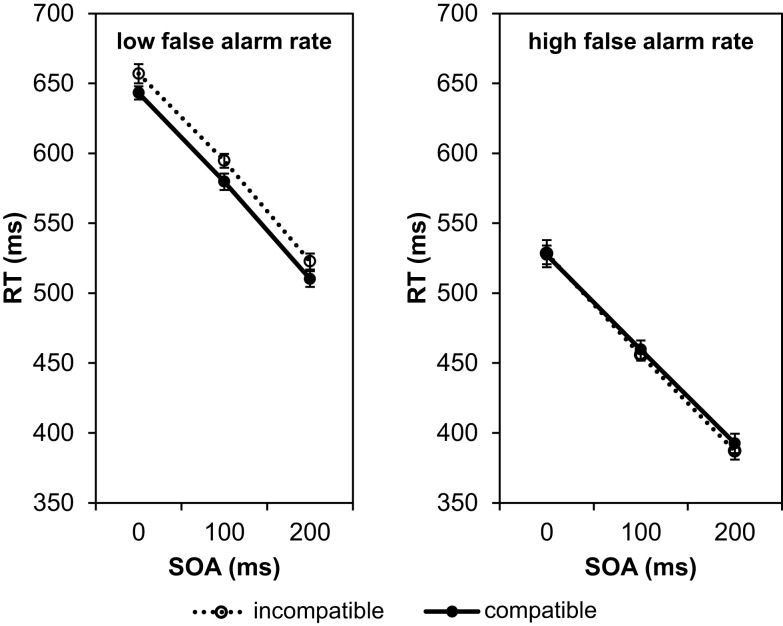
**Mean reaction times depending on SOA and compatibility conditions in Experiment 1**. The left graph shows the result for participants with a low rate of false alarms (less than 20% responses in No-Go trials), the right graph for participants with a high rate of false alarms (more than 20% responses in No-Go trials).

In a further step of the analysis we checked the participants’ false alarm rates (i.e., responses in No-Go trials). A high number of false alarms indicates that the participant did not follow the instructions and did not distinguish the Go stimuli from the No-Go stimulus. In that case the compatibility effect should disappear. On average the false alarm rate amounted to 17.46%, i.e., on average in 12.6 out of the 72 No-Go trials a response was wrongly executed. The inter-individual variance was large. Whereas 29 participants showed false alarms in less than 20% of the No-Go trials, some of the remaining 13 participants had false alarm rates of 40% and above, and one participant responded in all No-Go trials. Therefore, we recalculated the compatibility effects separately for participants with less than 20% false alarms (low false alarm rate) and participant with more than 20% false alarms (high false alarm rate; Figure 3).

A 3 × 2 × 2 ANOVA with SOA (0, 100, 200 ms) and compatibility (compatible/incompatible) as within-participant factors and the false alarm rate (low/high) as between-participants factor revealed that the effect of false alarm rates was significant, *F*(1, 40) = 18.44, *p* < 0.001, $\eta_{p}^{2}=0.32.$ Participants with a high false alarm rate responded considerably faster (mean RT = 458 ms) than participants with a low false alarm rate (mean RT = 584 ms). This underlines our assumption that participants with a high false alarm rate did not wait until they had fully identified the Go signal before they performed their response. As expected, the compatibility effect depended on the false alarm rate, *F*(1, 40) = 4.34, *p* = 0.044, $\eta_{p}^{2}=0.10.$ Participants with a low false alarm rate showed a compatibility effect of 14 ms, and those with a high false alarm rate did not show any compatibility effect (their responses to compatible trials were on average 3 ms slower than their responses to incompatible trials). Apart from the main effect of false alarm rates and the interaction of false alarm rates with compatibility, only the main effect of SOA was significant, *F*(2, 80) = 261.79, *p* < 0.001, $\eta_{p}^{2}=0.87.$ The main effect of compatibility did not reach significance, *F*(1, 40) = 1.92, *p* = 0.174, $\eta_{p}^{2}=0.05.$ All other interactions were not significant (*F*-values < 1).

In a further analysis, we tested if the compatibility effect developed with practice. To this end, we split the test phase in two halves. Only the data of the participants with a low false alarm rate were taken into account and were entered into a 2 (halves) × 3 (SOAs) × 2 (compatibility) ANOVA. We found a main effect of practice, *F*(1, 32) = 54.51, *p* < 0.001, $\eta_{p}^{2}=0.63.$ Responses in the second half were 79 ms faster than responses in the first half. Also the main effect of SOA, *F*(2, 64) = 238.01, *p* < 0.001, $\eta_{p}^{2}=0.88,$ and the main effect of compatibility could be confirmed, *F*(1, 32) = 8.40, *p* = 0.007, $\eta_{p}^{2}=0.21$ (large effect size according to Cohen, 1988). However, none of the interactions was significant. Most importantly, the compatibility effect was not affected by practice, *F*(1, 32) = 2.27, *p* = 0.14, $\eta_{p}^{2}=0.07.$ There was no evidence for an increase of the compatibility effect from the first to the second half. On the contrary, numerically the compatibility effect was larger in the first half of the test phase (18 ms) as compared to the second half (7 ms). For all other interactions the *F*-values were <1.

### Discussion

Experiment 1 clearly shows that the Go stimuli affected the preparation of the responses depending on their compatibility with the response effects. As long as participants had sufficiently processed the Go stimuli before executing the response, their RTs in effect-compatible trials were significantly shorter than in effect-incompatible trials. The compatibility effect disappeared for participants who ignored the Go stimuli. It is important to remember that the Go stimuli itself did not have any relationship to the two responses. Both stimuli appeared with equal frequency for each of the two responses. What made the Go stimuli compatible or incompatible was only their relationship to stimuli appearing after the execution of the responses. Therefore, the compatibility effect can only be explained by assuming that participants anticipated the effects of their responses during response preparation. Only if effect codes were active before response execution the compatibility between the Go stimuli and the future effects could affect the RTs. Consequently, dropping the effects in an additional experiment should abolish the compatibility effect. This was tested in Experiment 2.

In the experiment we could only find a main effect of SOA, confirming that participants indeed prepared the response during the SOA. The imperative stimuli informed the participants about the required response, and they used the time until presentation of the Go stimulus for response selection and programming. However, contrary to our original expectations, the SOA did not affect the compatibility effect. The compatibility effect did neither decrease nor increase with increasing SOA. Whereas a decrease of the compatibility effect would have indicated the involvement of effect anticipation in response selection, an increase of the compatibility effect would have supported the assumption that effects were anticipated for an already selected response. Both expectations were not confirmed by the data. A preliminary explanation might be that the present, rather simple task was not sensitive enough to provoke any interaction between SOA and compatibility. We therefore tested this further in Experiment 3.

A further interesting result was that practice during the test phase did not increase the compatibility effect. Apparently, then, the compatibility effect was fully developed from the beginning of the test phase. This supports the idea that the effect is based on the response effect relations as learned in the acquisition phase and not on relations that are only present in the test phase, such as the relationship between the imperative stimulus and the Go stimulus or between the Go stimulus and the response.

## Experiment 2

With the second experiment we wanted to assess whether the presence of the response effects in the test phase was crucial for the observed differences between effect-compatible and effect-incompatible trials. Alternatively, the association between responses and effects that was established during the practice phase might be sufficient for compatibility effects to emerge in the test phase, even if the effects are no longer present. We predicted that the compatibility effect would disappear when effects are no longer presented in the test phase. This result would further validate Experiment 1 by showing that the results were indeed due to the processing of the effect information during response preparation and not, e.g., to the imperative stimuli or Go stimuli and their assignment to the responses.

### Method

#### Participants

Thirty-six undergraduate students (29 female, 7 male) from the Department of Psychology at the University of Sunderland took part in the experiment. Participants had a mean age of 20.9 years (SD = 3.4). All participants received course credit for their participation. They had either normal or corrected to normal vision. Ethical approval was obtained from the departmental ethics committee.

#### Material and apparatus

These were identical to Experiment 1.

#### Design and procedure

The acquisition phase was the same as in Experiment 1. In the test phase, the only change consisted in the replacement of the effect stimuli by the word “correct.” Incorrect responses were followed by the word “incorrect.”

### Results

**Figure 4:**
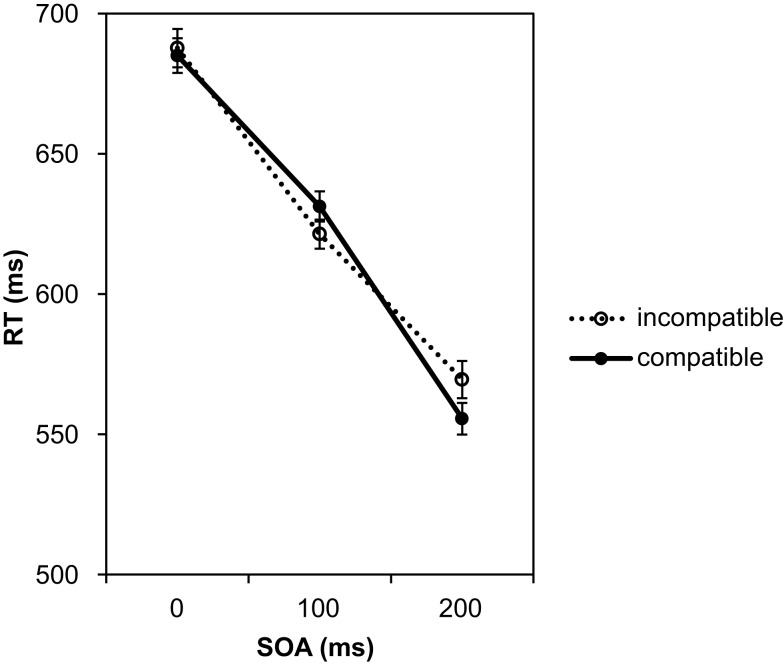
**Mean reaction times depending on SOA and compatibility conditions in Experiment 2**. Compatibility is defined in relation to the response effects presented in the acquisition phase for each of the responses.

Data were analyzed in the same way as for Experiment 1. Two of the participants exhibited exceptionally high error rates (about 50%, indicating random responses), and their data were thus discarded from the analysis. The remaining 34 participants exhibited an error rate of 3.7% which corresponds to the error rate in Experiment 1. The mean false alarm rate for the 34 participants was 10.17%. Only one participant had a false alarm rate above 20%, and we decided to also discard this participant’s data so that only those participants were included for which a compatibility effect would be most likely to occur. Figure 4 shows the mean RTs for effect-compatible and incompatible trials for each SOA.

Data were subjected to a 3 (SOA) × 2 (compatibility) ANOVA. The analysis only confirmed a significant effect of the SOA, *F*(2, 64) = 140.98, *p* < 0.001, $\eta_{p}^{2}=0.82.$ As in the first experiment, RTs decreased with the prolonged SOA. The RT difference between the 0 and the 200 ms SOA amounted to 124 ms. As expected, no significant effect of compatibility was found, *F*(1, 32) = 0.22, *p* = 0.643, $\eta_{p}^{2}=0.01.$ Also the interaction between both variables was not significant, *F*(2, 64) = 1.94, *p* = 0.152, $\eta_{p}^{2}=0.06.$ On average, for compatible trials responses were just 2 ms faster than for incompatible trials. Separate paired-samples *t*-tests for the three SOAs did not find any significant differences, 0 ms: *t*(32) = 0.28, *p* = 0.779, 100 ms: *t*(32) = 1.25, *p* = 0.220, 200 ms: *t*(32) = 1.72, *p* = 0.095.

In an additional analysis we split the test phase into two halves in order to identify a possible residual compatibility effect at the beginning of the test phase. The three-way ANOVA (test half, compatibility, SOA) only confirmed the results regarding compatibility and SOA reported above. Most importantly, there was no interaction between half and compatibility, *F*(1, 32) = 0.004, *p* = 0.950, $\eta_{p}^{2}=0.88<0.001.$ In fact, the difference between compatible and incompatible trials was only about 2 ms in both halves of the experiment.

### Discussion

Experiment 2 clearly confirms that the compatibility effect observed in Experiment 1 depended on the response effects. It only occurred in Experiment 1 where the response effects were present, but not in Experiment 2 where the differentiating effect stimuli were replaced by a single effect, the word “correct.” Up to response execution, everything was exactly the same in both experiments. Therefore, we can be very certain that the compatibility effect in Experiment 1 was not caused by any unknown relationship between the imperative stimuli, Go stimuli, and responses. This underlines that the compatibility effect observed in Experiment 1 is reliable evidence for the activation of effect codes during the preparation of a motor response.

We did not find any evidence that the compatibility effect would fade out in course of the test phase. The compatibility of the Go stimuli with the originally learned response effects did not affect the RTs from the beginning of the test phase. There was not even a numerical difference of the compatibility effects in the first and the second half of the test phase. Even when we only analyzed the first 30 responses of the test phase, no hint of a compatibility effect was found. Unfortunately, however, it is impossible to get reliable data for such small parts of the test phase because the number of repetitions per condition becomes too low. Within the first 30 trials there were only four responses per condition for each participant, and some of these data were missing due to errors and delayed responses. These factors might have obscured a possible residual compatibility effect at the beginning of the test phase. Nevertheless, it appears that participants realized very quickly that their responses only produced an unspecific effect in the test phase, and consequently they stopped anticipating the specific effects learned in the test phase.

The missing compatibility effect in Experiment 2 questions the interpretation of experiments in which the response effects did not appear after the responses in the test phase. For example, in Elsner and Hommel’s (2001) experiments as described above, the response effects were used as imperative stimuli in the test phase, but after the response no effect was presented. If participants stop anticipating effects if these effects are no longer appear after the response (as indicated by the present experiment), than the observed advantage of effect-compatible stimulus-response mappings might require a different interpretation. In another experiment, Cardoso-Leite et al. (2011) presented in half of the trials in the test phase no effects, and in the other half the effect stimuli appeared randomly after the responses. Their test phase was designed as detection task, and participants had to report if an effect stimulus was presented after the response. The authors found reduced sensitivity for response effects learned in the acquisition phase and explained this by effect anticipation; sensitivity for the expected stimulus is reduced, whereas the unexpected stimulus requires further processing. However, an alternative interpretation might be that participants stopped anticipating the previously learned effects and tried to learn and anticipate the new response effects, which could not be successful because of the random response effect assignment. The attempt to learn new effects might go along with the suppression of the old effects resulting in the reduced sensitivity.

## Experiment 3

In Experiment 1 we did not find any interaction between the SOA and the compatibility effect. As already mentioned, this might have been due to the simplicity of the task with just two responses. Under such conditions selection of responses and activation of effect codes might go too fast to find a compatibility × SOA interaction with our paradigm. In Experiment 3 we therefore used a more complex task with four responses and four effects. The larger number of response alternatives should slow down response selection and prolong the preparation period. In addition we extended the duration of the SOA up to 450 ms.

The second aim of Experiment 3 was to investigate the mechanisms that induce the compatibility effect in greater detail. The difference between compatible and incompatible trials could be caused by a facilitation of the response in compatible trials, an inhibition of the response in incompatible trials, or both. To differentiate between these alternatives, we introduced a new variation of the effect-incompatible Go stimuli: There were incompatible Go stimuli related to one of the response effects (incompatible/related) as in Experiment 1, as well as incompatible Go stimuli that had no relationship to any of the response effects (incompatible/unrelated). Assuming that the Go stimulus activates its corresponding representation in memory, in compatible trials the Go stimulus would activate a representation that is compatible with the anticipated response effect. In incompatible trials the Go stimulus would either activate a stimulus representation that is compatible with the effects of an alternative response (incompatible/related) or a stimulus representation which does not have any relationship to one of the response effects (incompatible/unrelated).

According to the ideomotor principle activation of effect codes leads to an automatic activation of the responses to produce these effects. Therefore, for compatible trials the compatible Go stimulus should eventually facilitate the response activation via the effect, while incompatible/related Go stimuli should result in the activation of competing responses and inhibit the required response. Compared to that, incompatible/unrelated Go stimuli should not facilitate or inhibit a competing response via the response effects. Hence, RTs should be shortest in compatible trials and longest in incompatible trials with incompatible/related Go stimuli, whereas incompatible/unrelated Go stimuli should result in intermediate RTs, due to reduced or absent response competition.

No difference between related and unrelated incompatible Go stimuli would be expected if there was only facilitation in compatible trials but no inhibition in incompatible trials. In both types of incompatible trials the Go stimulus would activate its representation in memory. However, that would not further activate codes of a related response effect in the case of incompatible/related Go stimuli and therefore would not lead to response competition.

In addition, we used Experiment 3 to test our paradigm further. Whereas in Experiment 1 objects were used as effects and hand postures as Go stimuli, in Experiment 3 hand postures were the effects, while objects were used as Go stimuli. If we found compatibility effects equivalent to Experiment 1, then we can conclude that these effects do not depend on the particular sequence of the Go and effect stimuli. Note also that the particular setting might increase the size of compatibility effect since, in line with the affordance concept (Gibson, 1979; Tucker and Ellis, 1998), the presentation of the objects as Go stimuli would activate the actions depicted in the effect stimuli.

### Method

#### Participants

Thirty undergraduate students of psychology at Liverpool Hope University took part in the experiment. Their mean age was 22.1 years (SD = 4.1). Fifteen of the participants were male and 15 female. Participants received course credit for their participation. Participants had either normal or corrected to normal vision. The experiment was approved by the departmental ethics committee.

#### Material and apparatus

**Figure 5:**
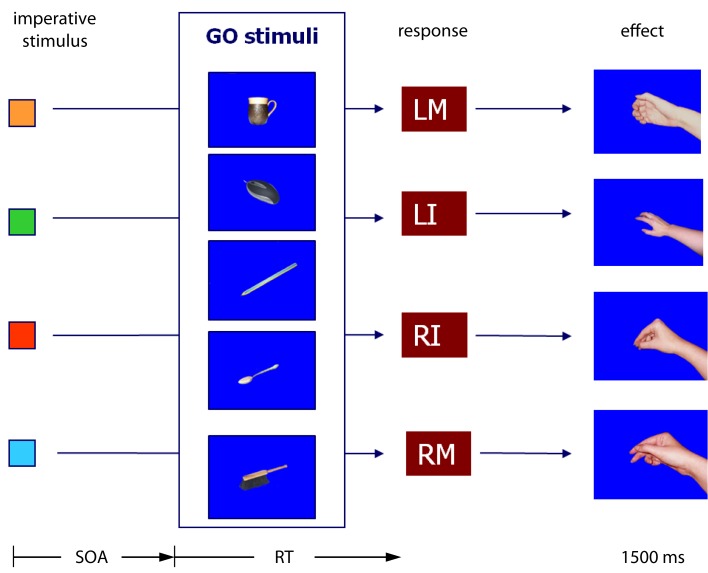
**Material and design of Experiment 3**. Cup, mouse, pen, and spoon are related to one of the hand postures used as effect stimuli. The pictures were used as compatible and incompatible Go stimuli. The hand brush is unrelated to all effect stimuli and was therefore incompatible to all of them. LM, left middle finger; LI, left index finger; RI, right index finger; RM, right middle finger. The SOA was 0, 150, 300, or 450 ms.

Participants had to choose between four responses. These were key-presses with the left and right middle and index fingers. The fingers were placed on the keys “Z,” “X,” “N,” and “M” on a standard QWERTY keyboard. Response effects were pictures of a right hand in the position of holding a coffee mug, a computer mouse, a pen, and a spoon assigned to the left middle finger, left index finger, right index finger, and right middle finger, respectively. In all cases, the hand was only shown without the corresponding objects. In the test phase the corresponding pictures of a coffee mug, a computer mouse, a pencil, and a spoon served as Go stimuli. Whereas these four Go stimuli had a relationship to one of the aforementioned hand postures, a fifth Go stimulus was a picture of a hand brush, which did not fit with any of the effect stimuli (see Figure 5). The No-Go stimulus was a picture of a hammer. As imperative stimuli in the test phase little squares in the colors orange, green, red, and blue were assigned in order to the left middle finger, left index finger, right index finger, and right middle finger.

#### Design and procedure

Basically, design and procedure of Experiment 3 were identical to that for Experiment 1. The acquisition and test phases followed exactly the same pattern as described above. There were again only 100 acquisition trials. That is, participants experienced on average only 25 instances of each response effect pairing. In the test phase, the colored squares served as imperative stimuli and the SOA between imperative stimulus and Go stimulus was 0, 150, 300, or 450 ms. The imperative stimuli (colored squares) were not occluded by the Go stimulus but stayed in the foreground. No-Go trials were indicated by the picture of a hammer. The No-Go stimulus always appeared after 600 ms in order to reduce the number of trials. All other details were identical to Experiment 1. Figure 5 illustrates the procedure.

In the Go trials, three types of Go stimuli were used. Go stimuli were either compatible with the effect (e.g., response with the left finger to produce a hand holding a coffee mug – picture of a coffee mug is a compatible Go stimulus), incompatible/related (e.g., response with the left finger to produce a hand holding a coffee mug – picture of a pencil is an incompatible Go stimulus, but related to the response with the right index finger producing a hand holding a pencil), or incompatible/unrelated (e.g., response with the left finger to produce a hand holding a coffee mug – picture of a hand brush is an incompatible Go stimulus that is unrelated to all effect pictures used in the experiment). In principle, four incompatible Go trials could have been generated, one with the hand brush and three with objects that corresponded to the effects of the other three responses. However, we decided not to use all possible combinations to avoid a bias in the experiment. If, on the one hand, all responses were combined with all Go stimuli with the same frequency, this had left us with 20% compatible trials and 80% incompatible trials, and the low frequency of compatible trials had likely worked against the compatibility effect. If, on the other hand, we had designed 50% of the trials as compatible and 50% as incompatible, each object picture had been combined more often with the response followed by the compatible effect than with all other responses. For compatible trials the Go stimulus had then biased the selection of the correct response, which had made it impossible to explain RT differences in terms of compatibility effects. Therefore, we limited the possible combinations of Go stimuli with the responses so that for each response there was exactly one compatible Go stimulus, one incompatible/related Go stimulus, and one incompatible/unrelated Go stimulus. For example, for a response with the left index finger only the coffee mug (effect-compatible), the pencil (effect-incompatible, related to the effect of the response with the right middle finger) and the brush (incompatible, unrelated to any of the other response effects) served as Go stimuli with equal frequency. In turn, for responses with the right index finger the coffee mug was the incompatible/related Go stimulus and the pencil the compatible stimulus. Following this design each of the four effect-related Go stimuli appeared with equal frequency for one response as effect-compatible Go stimulus and for another response as incompatible stimulus. The hand brush, as unrelated effect-incompatible Go stimulus, was used with the same frequency for all four responses.

The test phase consisted of 600 trials, with 480 Go trials and 120 No-Go trials. Among the Go trials, 160 trials were effect-compatible and 320 effect-incompatible (160 for each type of incompatible Go stimuli). All types of Go trials were presented at each SOA (0, 150, 300, 450 ms). Go trials and No-Go trials were randomly mixed. The full experiment lasted about 75 min.

### Results

Data from the acquisition phase were analyzed in order to check if participants had about the same learning experience with all responses and their effects. Given the total number of 100 trials, each response should have been executed 25 times. For each participant we calculated a response-use index, which was defined as the sum of the squared differences between 25 and the actual use for each of the four responses. An index of 0 would indicate that all responses had been used exactly 25 times. Indices varied between 20 and 1208. Whereas the small index of 20 showed that this participant deviated only by two to three responses from the ideal of 25 for each response, the highest index was due to using one of the responses only two times and another one 48 times. The mean response-use index was 182.1 (SD = 217.4). We decided to discard the two participants with the highest indices (1208 and 454) from the analysis since both had only rarely used at least one of the responses. In the test phase, the numbers of false alarms and erroneous responses were relatively low so that no other participants had to be excluded from further analysis: on average participants responded in 14.2% of the No-Go trials. Including outliers (RTs above 2000 ms) only 7.3% of the responses were erroneous responses.

**Figure 6:**
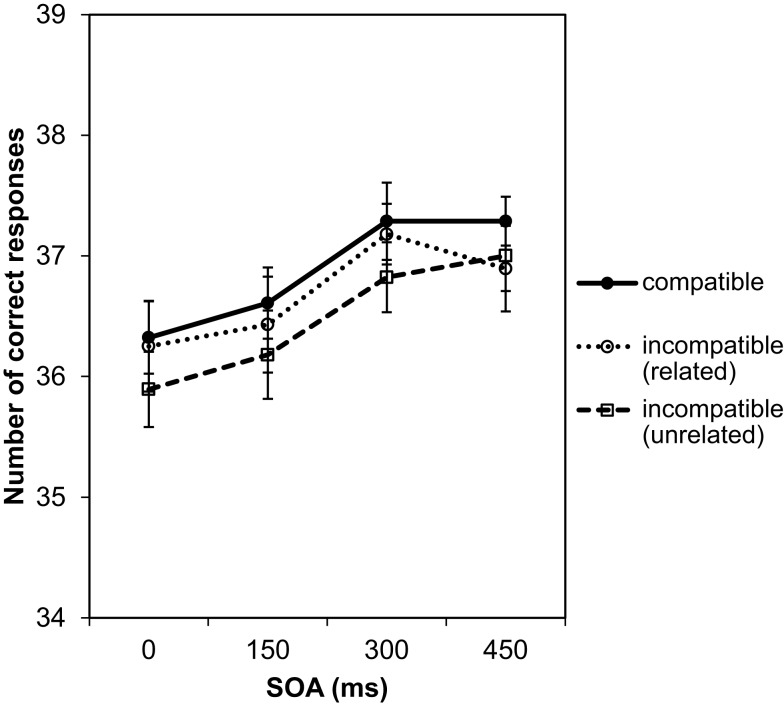
**Number of correct responses depending on SOA and compatibility**. The maximum number of correct responses for each experimental condition was 40. In counting correct responses, erroneous responses and slow responses with RTs over 2000 ms were excluded.

In analyzing the data of the test phase, first the number of correct responses per participant and condition was subjected to a 4 (SOAs) × 3 (compatibility conditions) repeated-measures ANOVA. There was only a significant main effect of SOA, *F*(3, 81) = 5.96, *p* = 0.001, $\eta_{p}^{2}=0.18$, indicating that the number of correct responses increased with longer SOAs (Figure 6). For effect-compatible trials, the number of correct responses was slightly higher than for incompatible trials. The lowest number of correct responses was observed for incompatible/unrelated trials (hand brush as Go stimulus). However, the compatibility effect was not significant, *F*(2, 54) = 1.30, *p* = 0.280, $\eta_{p}^{2}=0.05,$ nor was the interaction between SOA and compatibility, *F*(6, 162) = 0.12, *p* = 0.994, $\eta_{p}^{2}=0.004.$

**Figure 7:**
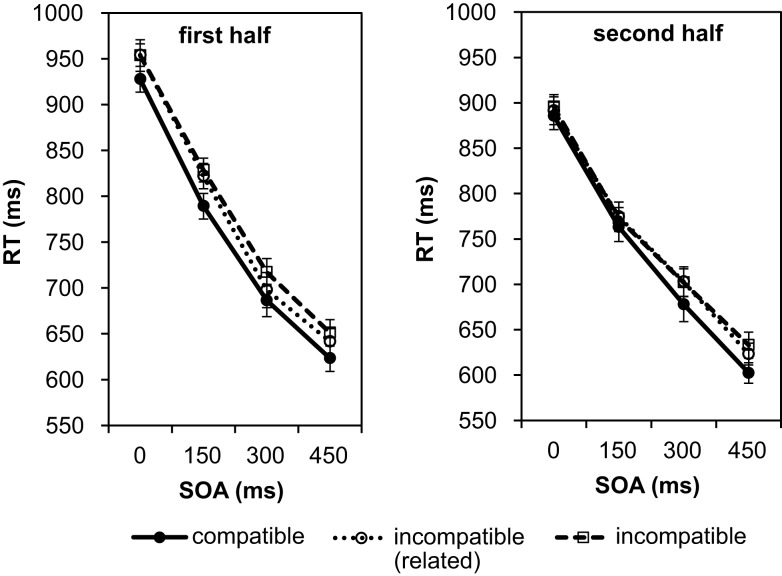
**Mean reaction times depending on SOA and compatibility in Experiment 3**. The data are shown separately for the first half of the test phase (left graph) and the second half of the test phase (right graph).

In a second step the RTs were analyzed. As in the previous analyses, we split the experiment into two halves to check for possible practice effects on the RTs (see Figure 7).

The individual means per experimental condition were entered into a 2 (halves) × 4 (SOAs) × 3 (compatibility conditions) repeated-measures ANOVA. Practice from first to second half led to a small, significant facilitation of responses by 31 ms, *F*(1, 27) = 4.50, *p* = 0.043, $\eta_{p}^{2}=0.14.$ There was also a significant effect of SOA, *F*(3, 81) = 391.22, *p* < 0.001, $\eta_{p}^{2}=0.94.$ As in the first two experiments, and in line with the error analysis above, the responses became not only more accurate but also faster with increasing SOA. At the 450 ms SOA responses were 289 ms faster as compared to the 0 ms SOA. Again, this reflects that participants used the SOA to prepare the response. The SOA effect was 35 ms smaller in the second half as confirmed by the significant interaction between SOA and the halves of the experiment, *F*(3, 81) = 2.94, *p* = 0.038, $\eta_{p}^{2}=0.1.$ Most importantly, we found a significant effect of compatibility, *F*(2, 54) = 8.45, *p* = 0.001, $\eta_{p}^{2}=0.24$ (large effect size according to Cohen, 1988). Pairwise comparisons using the Bonferroni correction revealed that RTs under the effect-compatible condition were significantly shorter than under both incompatible conditions (incompatible/related: difference = 19 ms, *p* = 0.026; incompatible/unrelated: difference = 25 ms, *p* < 0.001). The difference between the two incompatible conditions was not significant, *p* > 0.999. Thus, as in Experiment 1, the relationship between Go signal and response effect clearly modulated the RTs. Taking the analysis of the number of correct responses into account we can exclude a speed-accuracy trade-off in explaining the observed difference. However, similar to Experiment 1, the compatibility effect did not depend on the SOA, *F*(6, 162) = 0.09, *p* = 0.997, $\eta_{p}^{2}=0.003.$ Furthermore, the compatibility effect did not change with practice, *F*(2, 54) = 0.41, *p* = 0.665, $\eta_{p}^{2}=0.02.$ The numerical data in Figure 7 suggest that in the second half of the experiment the compatibility effect increased with increasing SOA. However, this was not confirmed by the statistical analysis as the three-way interaction was not significant, *F*(6, 162) = 0.36, *p* = 0.905, $\eta_{p}^{2}=0.01.$ Also a separate ANOVA for the second half of the test phase did not reveal an interaction between compatibility and SOA, *F*(6, 162) = 0.21, *p* = 0.972, $\eta_{p}^{2}=0.01.$ Only the main effects of compatibility, *F*(2, 54) = 3.24, *p* = 0.047, $\eta_{p}^{2}=0.11,$ and of SOA, *F*(3, 81) = 156.67, *p* < 0.001, $\eta_{p}^{2}=0.85,$ were significant.

### Discussion

The third experiment demonstrates three important points: First, the compatibility effect between the Go stimulus and the response effect, as observed in Experiment 1, did not depend on the specific stimulus material. Under more complex conditions, and using different stimuli, we have observed the same effect again. Whereas we had originally expected a larger compatibility effect than in Experiment 1, this was not the case. This might be due to the fact that with the chosen design we had partially worked against an increase of the compatibility effect. Compatible trials had a lower frequency than incompatible trials (one-third against two-thirds of trials) whereas in Experiment 1 compatible and incompatible trials were equally frequent.

Second, also under the more complex conditions and extended SOAs, the compatibility effect was not affected by the SOA. This does not correspond to our original predictions. However, the present results make it unlikely that effect anticipation is the prerequisite for response selection, at least under the conditions of our experiment. Had this been the case, then in particular early compatible Go stimuli should have facilitated the responses, and the compatibility effect should have decreased with increasing SOA. The data do not show any tendency for such a pattern.

Third, it is important to note that there was no difference between the two types of incompatible trials. This indicates that only compatible Go stimuli facilitated the anticipation of the response effect, but incompatible Go stimuli did not seem to inhibit the anticipation of the effect or to activate competing responses. We assume that the representations activated by the Go stimuli do not directly activate representations of corresponding response effects. However, if representations of the response effects are anticipated depending on a selected response, compatible Go stimuli might contribute to further activation of these representations whereas incompatible Go stimuli remain without effect. This resembles very much our findings with the flanker task (Ziessler and Nattkemper, 2002, 2011; Ziessler et al., 2004). Compared to neutral stimuli, the presentation of response effects flanking the imperative stimulus facilitated the response only if the flanking stimuli were the effect of the required response. But also in that study, no inhibition was found when the effects of competing responses were presented.

## General Discussion

The present experiments provide evidence for effect anticipation using a new paradigm that does not require the presentation of the effects itself in the planning phase of the response. Participants were instructed to prepare a particular response, but to withhold its execution until the stimulus configuration would allow it. This is a relatively natural situation. Very often we are prepared to do something, but we have to wait for the suitable conditions before we can actually do it, for example waiting for the green traffic light before we can move the car forward. In the present experiments the execution conditions were defined by Go stimuli. The Go stimuli themselves had no relationship to the responses. Therefore, if the selection of a response and its preparation would depend only on the stimuli presented before response execution, the Go stimuli should not make any difference between the responses and should not affect response execution. In contrast to this assumption, Experiments 1 and 3 showed that events which occurred after the execution of the response also played a role in the reaction time interval. Depending on their relationship to the learned effects of the responses, the Go stimuli were either compatible or incompatible with the given response. The small, but consistent RT differences between effect-compatible and incompatible trials can only be explained by assuming that the Go stimuli interacted with the effect codes. In other words, effect codes must have been active in advance of response execution. Thus, we have found clear evidence for effect anticipation as part of response preparation without presenting the effects themselves during response preparation and also without any feature overlap between responses and effects.

Interestingly, in the present experiments the effects were completely irrelevant for the responses. In the test phases the identity of the required response was fully determined by the imperative stimulus. Participants could execute fast and accurate responses without taking the effects into account. However, the results show that the response effects were anticipated. Apparently, the anticipation of effects is a very basic process. When we plan a motor response or motor action we anticipate the effects that the response or action will cause in the environment, at least if the effects are attended to (Ziessler et al., 2004).

The experiments also show that the cognitive systems associated with response planning are very flexible in learning response effects and in applying this knowledge in motor control. The effects that we examined in the present experiments were completely arbitrary. A small number of acquisition trials was sufficient to create response effect relations that affected the RTs in the following test phase. In Experiment 3 participants had only about 25 repetitions of each response for acquiring the response effects in the acquisition phase. Note that the participants were not explicitly instructed to learn the effects. Nevertheless, we found compatibility effects early on in the test phase and, as the comparisons between the first and second half of the test phase indicated, these compatibility effects did not increase with further practice. Thus, on the one hand it seems that participants learn response effect relations very quickly (see also Wolfensteller and Ruge, 2011). On the other hand, our experiments also show that participants drop their response effect knowledge immediately if the effects are no longer valid. In Experiment 2, participants were given the same opportunity as participants in Experiment 1 to learn the response effects. However, in the test phase they clearly did no longer anticipate the learned effects after they detected that the effects would not appear after the responses.

The compatibility effect did not interact with the SOA. Originally we had expected that the compatibility effect should either decrease or increase with the SOA between the imperative stimulus and the Go stimulus. The data show that this was not the case. In Experiments 1 and 3 the compatibility effect was very stable over the SOA variation. Only the second half of the test phase of Experiment 3 showed a numerical increase of the compatibility effect with the SOA. However, this increase was not statistically significant. In two additional experiments not reported here we repeated Experiments 1 and 3 without the acquisition phase. Participants could only learn the response effects in the test phase. The idea behind this variation was that the process of effect anticipation might change with increasing practice from a more goal-oriented to a more stimulus-driven mode (Ruge et al., 2012). Thus, after extensive practice participants might just respond to the stimuli but not use effect anticipation for response control. The results confirmed that participants learned the effects very quickly. However, also if the participants could only learn the response effects in the test phase, the compatibility effect did not depend on the SOA.

**Figure 8:**
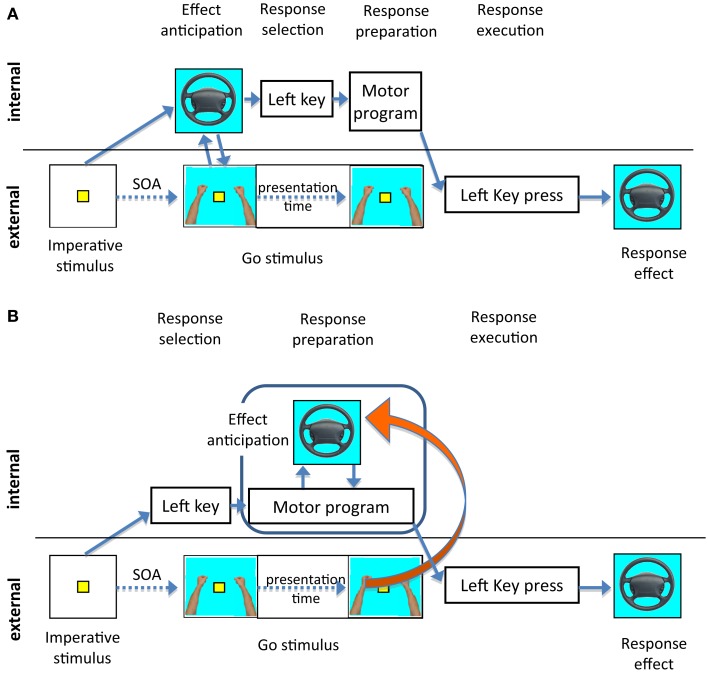
**Illustrations of alternative explanations of the compatibility effect and its relationship to the SOA**. The dotted line to the right of the Go stimulus illustrates the presentation of the Go stimulus. **(A)** The imperative stimulus directly activates the representation of the effect. Depending on the activated effect codes the response is selected. A compatible Go stimulus would contribute to the activation of the same effect code and therefore facilitate the selection of the response, provided the Go stimulus was presented before the completion of the response selection. **(B)** The response is selected depending on the imperative stimulus. After response selection the effect of the response is anticipated to enable the monitoring of the response and error detection. The anticipation is supported by a compatible Go stimulus throughout response preparation.

There are two ways to interpret the general stability of our compatibility effects across different SOAs. First, it could be assumed that response selection in the present experiments indeed required the activation of effect codes as assumed by strong versions of the ideomotor theory. Following this approach, response selection occurs through the activation of effect codes (Lotze, 1852; Harleß, 1861; Hommel et al., 2001). Effect codes would then be activated early in the process and would remain active throughout response preparation. Consequently, the Go stimulus in our paradigm could in principle interact with the anticipated effect at all SOAs. However, one might expect the strongest compatibility effects for the shortest SOA, that is, when imperative and Go stimuli are presented together: In this case the presentation of an effect-compatible Go stimulus could prime the anticipation of the response effect and facilitate the response as observed in Experiment 3. Figure 8A illustrates this scenario.

Whilst this explanation works very well for the short SOAs, it is more difficult to explain why the compatibility effect would not decrease at longer SOAs: when the effect codes are already fully activated and responses are selected, the impact of the Go stimulus should become weaker. One way to explain compatibility effects at longer SOAs within this first framework might be to assume an additional mechanism: Based on the anticipated effect, participants might facilitate the processing of effect-compatible Go stimuli presented at long SOAs, compared to trials where the Go stimulus is different from the predicted. This means, not only would the Go stimulus prime effect anticipation, but also the anticipated effect would prime the processing of the effect-compatible Go stimulus.

A second explanation assumes that effects are anticipated based on an already selected response. Figure 8B illustrates this scenario, which is also supported by a number of findings in other experiments. Using a flanker task, we found evidence that effect codes were activated about 150–300 ms after presentation of the imperative stimulus (Ziessler and Nattkemper, 2011). In these experiments the effects of the response were presented as flanking stimuli together with the imperative stimulus. The strongest flanker effects were found if the effect flankers followed the imperative stimulus whereas effect flankers presented in advance of the imperative stimulus had no effect. Further, Nikolaev et al. (2008) could confirm the assumption that effect anticipation takes place after response selection using event related potentials (ERPs) in the same paradigm. In their experiment, ERPs evoked by effect-incompatible flankers differed from those evoked by other flankers in an early perceptual component indicating an inhibition of perceptual representations incompatible with the response, and in later components related to stimulus evaluation and response detection. In addition, the time difference between the lateralized readiness potentials and the onset of the response indicated that also the processes of motor execution were affected by incompatible flankers.

If effects are anticipated for an already selected response, then one might expect that, in the present experiments, the interaction between the Go stimuli and the anticipation of the response effects should be most pronounced at later stages of response preparation, i.e., for the longer SOAs. However, given that in the present experiments, the Go stimulus remained on the screen until the response key was pressed, even at short SOAs the Go stimulus could have interacted with the anticipation of response effects at any time before execution. In a third additional experiment not reported here, we had therefore presented the Go stimulus for a 100 ms period only. Again, there was a main effect of compatibility, but even with this restricted duration of the Go stimulus, the compatibility effect did not reduce at the longer SOAs. It is thus conceivable that the present methodology does not allow to narrow down the precise time point at which the compatibility effects emerge. The paradigm indeed requires that participants process the Go stimulus and keep its representation active throughout response preparation because this is the information indicating that they should finally execute the key-press. If this is correct then the Go signal could affect the anticipation of the response effect at any stage of response preparation.

Even though both interpretations are not exclusive, we consider our findings more in line with the second interpretation. In particular the fact that the early Go stimuli did not induce stronger effects than the late Go stimuli is difficult to align with the idea that effect anticipation is a prerequisite of response selection. This is, of course, not an argument against the strong version of the ideomotor principle as such. We fully agree with Herwig et al. (2007) and Keller et al. (2006) who distinguished between stimulus-based (“response mode”) and intention-based actions (“intention mode”). In the intention mode, participants develop stronger action effect bindings and might use the effects in turn to select the actions. The test phase of our experiments was a choice reaction task, i.e., participants were in the response mode and did not need the response effects for response selection. Presumably they only used the effect anticipation for subsequent processing. This might also explain why we could not find any evidence for effect anticipation in Experiment 2 where the response effects were no longer presented, whereas other authors reported effect anticipation without physical presence of the response effects in the test phase. For example, Kühn et al. (2010) used a free-choice task in both, the acquisition and the test phase. Under this condition response effects learned in the acquisition phase led to differences in brain activations in the test phase where the effects were not presented. If we assume that participants in the free-choice task are more likely to act in the intention mode, then the different outcomes of their study and of our Experiment 2 become intelligible. To make sense of the free-choice task participants begin to intend the production of one of the response effects. This does not necessarily change when the effects do not appear in the test phase. Therefore, with the present experiments we do not want to exclude that the anticipation of response effects plays a role in response selection. However, in our paradigm the other functions of effect anticipation were likely more prominent.

In conclusion, with the present experiments we present a new paradigm providing more clear-cut evidence for the anticipation of response effects during response preparation than previously available. Effect anticipation was demonstrated via priming by another stimulus presented during response preparation. The paradigm does not include any direct activation of effect codes by external stimuli and therefore overcomes a possible objection to earlier studies.

## Conflict of Interest Statement

The authors declare that the research was conducted in the absence of any commercial or financial relationships that could be construed as a potential conflict of interest.
